# Methylarginine levels and their impact on vascular aging: a systematic review

**DOI:** 10.1530/VB-25-0004

**Published:** 2025-12-23

**Authors:** Eduardo Eric Almeida do Carmo, Celia Cristina Diogo Ferreira, Roberta Melquiades Silva de Andrade

**Affiliations:** Institute of Food and Nutrition, Federal University of Rio de Janeiro, Macaé, Brazil

**Keywords:** vascular aging, methylarginines, ADMA, SDMA, biomarkers, endothelial dysfunction

## Abstract

Vascular aging is a multifactorial process characterized by structural and functional changes that compromise endothelial homeostasis and increase the risk of cardiovascular disease. Among the factors involved in this process, methylarginines, such as asymmetric dimethylarginine (ADMA), symmetric dimethylarginine (SDMA), and NG-monomethyl-L-arginine (L-NMMA), stand out. These negatively modulate nitric oxide (NO) bioavailability, compromising endothelial function. This systematic review aimed to investigate the relationship between vascular aging and methylarginine levels, considering their influence on endothelial dysfunction and its impact on human health. The systematic search was conducted in scientific databases, resulting in the inclusion of four studies: three observational studies in humans and one experimental study *in vitro*. The findings demonstrated that elevated levels of ADMA, SDMA, and L-NMMA are associated with the progression of endothelial dysfunction, increased cardiovascular risk, and cognitive impairment in the elderly. The *in vitro* study reinforced this evidence by demonstrating that increasing concentrations of ADMA accelerate endothelial cell senescence, reduce telomerase activity, and decrease NO production. Interpretation of the results should consider the methodological limitations of the included studies, but the findings reinforce the role of methylarginines as potential biomarkers of vascular aging and highlight the need for further investigations exploring therapeutic strategies to minimize their deleterious effects.

## Introduction

The global demographic transition, characterized by increased longevity, poses significant public health challenges, especially regarding the cardiovascular system (1, 2). Physiological aging is associated with structural and functional changes, such as increased arterial stiffness and endothelial dysfunction (3, 4). These changes directly contribute to the prevalence of cardiovascular diseases, the leading causes of morbidity and mortality in the elderly population (5, 6, 7). Understanding these mechanisms is fundamental for developing effective therapeutic interventions and promoting healthy aging (8).

The endothelium plays a crucial role in vascular health, with endothelial dysfunction being an early indicator of age-related heart disease (9, 10). This dysfunction is often linked to reduced bioavailability of nitric oxide (NO), an essential vasodilator (11, 12). NO production is regulated by the enzyme endothelial nitric oxide synthase (eNOS) and can be compromised by the presence of endogenous inhibitors, such as methylarginines (ADMA, SDMA, and L-NMMA), which compete with L-arginine for eNOS (13).

Despite growing knowledge about vascular physiology, the specific relationship between methylarginine accumulation and vascular aging remains poorly documented and understood (14, 15). The impact of these compounds on endothelial dysfunction and arterial stiffness, under both physiological and pathological conditions, remains a gap in the literature. Therefore, this work proposes a systematic review to investigate the role of methylarginines as mediators of vascular aging, exploring the relationship with endothelial dysfunction and human health outcomes.

## Methods

This study is a systematic review conducted according to the PRISMA 2020 (Preferred Reporting Items for Systematic Reviews and Meta-Analyses) guidelines. The study identification, screening, eligibility, and inclusion steps were rigorously followed. The literature search was conducted in the PubMed, Scopus, Medline, Embase, Virtual Health Library, and SciELO databases. The review protocol was not registered on platforms such as PROSPERO.

### Eligibility criteria

Eligibility criteria were established before the search began, considering only original studies investigating the relationship between methylarginines – including ADMA, SDMA, or L-NMMA – and markers of vascular aging, such as endothelial function, arterial stiffness, cellular senescence, and cardiovascular mortality. Studies published between 2014 and 2024, available in English, Portuguese, or Spanish, with observational, experimental, or clinical trial designs were included, provided they presented direct measurement of at least one plasma or intracellular methylarginine. Articles addressing clinical conditions capable of independently altering methylarginines, such as advanced renal failure, sepsis, or cancer, were excluded, as were narrative reviews, editorials, letters, theses, dissertations, secondary analyses, and duplicate studies.

### Search strategy

The search strategy used a combination of keywords with the Boolean operators ‘AND’ and ‘OR’. The following combinations were applied: ‘vascular aging’ AND ‘methylation’, ‘endothelial function’ AND ‘vascular senescence’, ‘endothelial senescence’ OR ‘vascular senescence’ AND ‘methylation’, ‘methylarginines’ AND ‘NO’, ‘methylarginines’ AND ‘endothelial function’. Specific filters for each database were used, including publication period.

### Study selection process

The study selection process was carried out in successive stages. Initially, all records identified in the databases were imported into an Excel spreadsheet, where they were organized according to author, year of publication, country of origin, study population, and main results. At this stage, duplicate studies were removed. Next, two reviewers independently screened the titles and abstracts to identify articles directly related to the theme of this review. Potentially relevant studies were then fully evaluated in the eligibility stage, in which compliance with the previously established criteria was verified. Those that directly addressed the scope of the investigation were included for final analysis. Data extraction was conducted independently by two reviewers using a standardized form containing information on authorship, year and country, population characteristics, design, methods of measuring methylarginines (such as LC-MS/MS and ELISA), vascular markers evaluated, and main findings related to vascular aging. Considering the methodological heterogeneity among the studies, as well as the differences between populations and outcomes, it was not possible to perform a meta-analysis; therefore, a qualitative synthesis was chosen, emphasizing physiological mechanisms, consistency of results, and methodological limitations.

### Data extraction and synthesis

For each selected study, data such as authors, year of publication, country of origin, study population, and main findings were extracted. The data synthesis is qualitative, focusing on the molecular, biochemical, and physiological mechanisms that explain the relationship between vascular aging and methylarginine levels, as well as their impact on endothelial markers.

### Risk of bias

The risk of bias assessment of the included studies was performed using appropriate tools for each type of methodological design. The three observational studies were assessed using the NIH quality assessment tool for observational cohort and cross-sectional studies (NHLBI, 2017), while the *in vitro* experimental study was analyzed using an adaptation of SYRCLE’s risk of bias tool (Hooijmans *et al.* (16)), considering specific criteria for cellular experiments.

## Results and discussion

**Figure 1:**
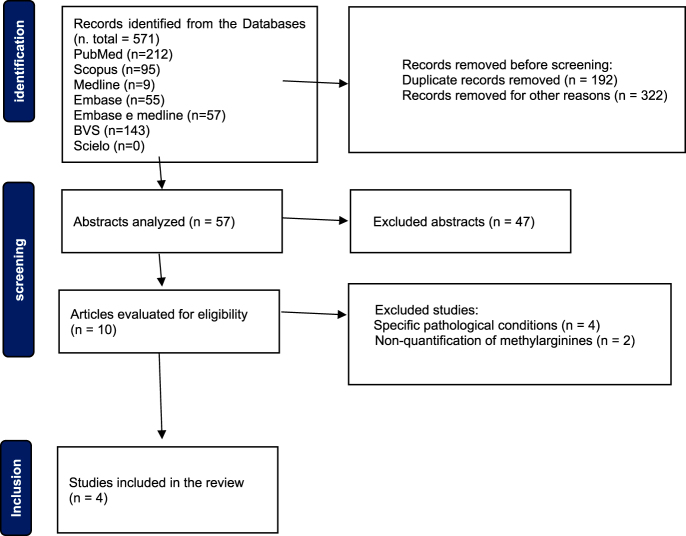
PRISMA 2020 flow diagram of the study selection process. Flowchart illustrating the identification, screening, eligibility, and inclusion phases of studies for the systematic review.

The study selection process is illustrated in Fig. 1, following the PRISMA 2020 guidelines.

The qualitative synthesis of this systematic review of the four included studies provides insights into the role of methylarginines in vascular aging (Table 1). The studies analyzed provide evidence that alterations in L-arginine metabolism, specifically the increase in its endogenous inhibitors (methylarginines), are associated with endothelial dysfunction, cognitive decline, and a higher risk of cardiovascular mortality. The small number of eligible studies highlights the knowledge gap in the literature, reinforcing the relevance of this research.

**Table 1:** Results of original studies on methylarginines and vascular aging.

Study	Country of study	Objective	Sample	Mean age, years	Main findings
Klawitter *et al.* (17)	USA	To investigate the relationship between relative L-arginine deficiency and endothelial dysfunction throughout the different stages of the menopausal transition in women	129 women divided into different menopause groups: Premenopause: 36 Early perimenopause: 16 Late perimenopause: 21 Early postmenopause: 21 Late postmenopause: 35	Prepmenopause: 33 ± 7 Onset of perimenopause: 49 ± 3 End of perimenopause: 50 ± 4 Onset of postmenopause: 55 ± 3 Late postmenopause: 61 ± 4	Increased ornithine in postmenopausal women compared to premenopausal women Increased L-NMMA in postmenopausal women compared to pre- and perimenopausal groups Decreased L-arginine/L-NMMA ratio in postmenopausal women compared with pre- and perimenopausal women Progressive reduction of L-arginine during perimenopause with recovery in postmenopause ADMA showed no significant change between menopausal stages
Staniszewska *et al.* (19)	UK	To determine the relationship between SDMA levels and all-cause mortality, in addition to assessing disease severity in patients with symptomatic PAD	238 patients with PAD and ankle-brachial index <0.8 (ABI)	69 (range: 62–75)	L-arginine did not change between groups SDMA was higher in the group of people who died ADMA was higher in the group of people who died
McEvoy *et al.* (18)	Australia and UK	To measure serum concentrations of vascular risk factors, ADMA and SDMA, in a representative sample of older adults and determine their associations with objective and subjective memory impairment	483 elderly adults	55–85 (IQR: 60–70)	Increased SDMA (Q4) was associated with impairment of objective memory Increased SDMA (Q4) was associated with impaired subjective memory ADMA (in all quartiles) was associated with subjective memory impairment No statistically significant association was found between L-arginine levels or the L-arginine/ADMA ratio and memory impairment, either objective or subjective
Scalera (25)	Germany	To investigate how ADMA accelerates cellular senescence in HUVEC, exploring the molecular mechanisms involved	HUVEC	Not applicable	SA-β-gal increased in a dose-dependent manner upon ADMA administration Telomere length was reduced proportionally to the increase in ADMA Telomerase activity was reduced in all groups treated with ADMA, with a more pronounced reduction in the 100 μM group NO production (measured by NOx) decreased in a dose-dependent manner

SDMA, symmetric dimethylarginine; PAD, peripheral arterial disease; ADMA, asymmetric dimethylarginine; HUVEC, human endothelial cells; IQR, interquartile range; SA-β-gal, senescence-associated beta-galactosidase.

### Risk of bias assessment

The study by Klawitter *et al.* (17), which investigated the relationship between L-arginine and endothelial dysfunction in menopause, presented a moderate risk of bias. Although it used flow-mediated dilation (FMD) as the primary outcome, a validated and widely used method for assessing endothelial function, the study did not apply blinding of the evaluators, which may have introduced bias in the measurement of results. In addition, although it adjusted for some confounding variables, it did not take into account relevant metabolic factors, such as diet and physical activity, which can significantly influence L-arginine levels and NO bioavailability. Thus, there is uncertainty about the actual extent of the association between L-arginine and endothelial dysfunction in the different stages of menopause.

The 2014 study by McEvoy *et al. *(18), which evaluated the relationship between methylarginines and memory in older adults, also presented a moderate risk of bias. The study used plasma biomarkers measured by mass spectrometry (LC-MS/MS), ensuring high precision in the measurement of ADMA and SDMA concentrations. Memory was assessed using both objective tests and a subjective scale, providing a more comprehensive analysis of the participants’ cognition. However, a critical point was the lack of information regarding the blinding of evaluators in the application of cognitive tests, which may have influenced the interpretation of the results. In addition, metabolic and behavioral variables that affect both cognition and methylarginine levels were not fully adjusted, leaving room for outcome distortion. Although the findings are relevant, the lack of control for these factors requires caution in extrapolating conclusions.

The study by Staniszewska *et al.* (19), which investigated the relationship between SDMA and mortality in patients with peripheral arterial disease, was classified as having a low risk of bias. The long follow-up period of approximately 7 years strengthens the reliability of the findings, allowing for a better assessment of the relationship between baseline SDMA and ADMA levels and clinical outcome. Biomarkers were quantified by HPLC-MS/MS, a highly accurate method, and multivariate analysis considered important clinical variables, reducing the risk of confounding bias. The low loss rate during follow-up reinforces the robustness of the study. However, the single measurement of SDMA and ADMA may be a limitation, as it does not allow for the assessment of variations over time and their dynamic relationship with the progression of peripheral arterial disease. Nevertheless, methodological consistency and statistical rigor support the validity of the findings.

The *in vitro* experimental study presented a moderate to high risk of bias. Although well-established biochemical methods were used to assess cellular senescence (SA-β-gal), telomerase activity, and NO synthesis, some methodological weaknesses compromise the interpretation of the results. The absence of clear randomization in the allocation of experimental groups raises concerns about the distribution of cells treated with different concentrations of ADMA. In addition, there is no indication that the experimenters were blinded to the experimental conditions, which may have introduced bias during the conduct of the trials. Another important limitation is the lack of detail on the number of biological and technical replicates, which may affect the reproducibility of the findings. As *in vitro* studies serve as support for mechanistic hypotheses, these limitations do not completely invalidate the results but require replication in additional models for greater reliability.

Considering the methodological limitations identified, the findings of this review should be interpreted with caution. These methodological differences should be taken into account in the analysis of the results and reinforce the need for future investigations using more rigorous methodologies to further explore the relationship between methylarginines and vascular aging.

### Methylarginines and vascular aging

This systematic review included four studies investigating the relationship between methylarginines, aging, and vascular health. The analyzed studies provide evidence that changes in L-arginine metabolism, specifically the increase of its endogenous inhibitors (methylarginines), are associated with endothelial dysfunction, cognitive decline, and a higher risk of cardiovascular mortality. The small number of eligible studies highlights a significant knowledge gap, reinforcing the relevance of this investigation.

### The role of methylarginines in vascular aging and endothelial dysfunction

The results of this systematic review reinforce the hypothesis that the accumulation of methylarginines, especially ADMA and SDMA, acts as a key mediator in the progression of vascular aging (20, 21). Endothelial dysfunction, an initial manifestation of many age-related cardiovascular pathologies, appears to be directly modulated by these endogenous eNOS inhibitors (22). The evidence that elevated levels of ADMA and SDMA are associated with increased mortality in patients with peripheral arterial disease (19) highlights the clinical relevance of these biomarkers. This finding suggests that methylarginine-mediated endothelial dysfunction may not only be a marker but also an active contributor to adverse clinical outcomes in aging populations.

The correlation between increased methylarginines and cardiovascular vulnerability in postmenopausal women, as observed by Klawitter *et al.* (17), is particularly relevant to the field of aging. The decrease in the L-arginine/L-NMMA ratio in these individuals indicates an imbalance in NO metabolism (23), a central mechanism for vascular aging. This imbalance leads to a reduction in NO bioavailability and, consequently, to a progressive deterioration of endothelial function, a hallmark of both physiological and pathological aging (24).

### Molecular mechanisms and systemic implications

The *in vitro* study by Scalera *et al.* (25) provides a solid mechanistic basis for the observational findings. By demonstrating that exposure of endothelial cells to ADMA accelerates cellular senescence, shortens telomeres, and reduces telomerase activity, the study establishes a direct link between methylarginines and cellular aging processes. Endothelial senescence compromises the vessel’s ability to adapt to hemodynamic stimuli and repair damage, contributing to arterial stiffness and atherosclerosis, two characteristics of vascular aging (13).

In addition to direct cardiovascular implications, the review also points to the systemic impact of methylarginines. The study by McEvoy *et al.* (18) associated elevated SDMA levels with memory impairment in the elderly. This finding is significant, as endothelial dysfunction, mediated by methylarginines, can impair cerebral circulation, leading to chronic ischemia and contributing to cognitive decline (25). This connection between vascular health and neurodegeneration underscores the importance of a holistic approach to aging, considering biomarkers such as methylarginines in different clinical contexts (26, 27).

### Limitations and future research directions

Although the qualitative synthesis of the studies supports a fundamental role for methylarginines in vascular aging, it is crucial to acknowledge the methodological limitations encountered, such as the moderate risk of bias in some observational studies and the heterogeneity of the studied populations (16, 28). The scarcity of randomized clinical trials and high-quality prospective studies on the topic represents a notable gap in the literature (14).

## Conclusion

This review showed that methylarginines, such as ADMA, SDMA, and L-NMMA, play an important role in vascular aging, influencing the bioavailability of NO and, consequently, endothelial function. However, the studies analyzed indicate that the concentration of these methylarginines is modulated by a variety of factors, including hormonal changes, such as those that occur in the different phases of menopause, inflammatory states, metabolic dysfunctions, and the progression of physiological aging itself. These variables influence processes such as arginine methylation, DDAH enzyme activity, and renal excretion of dimethylated forms, directly impacting the circulating concentration of these molecules.

The reviewed findings suggest that changes in the methylarginine profile, especially in the L-NMMA/arginine ratio, may be associated with endothelial dysfunction in certain conditions, such as in the different phases of menopause. In addition, the literature points out that increased concentrations of these molecules may be related to the progression of cardiovascular diseases, intensifying oxidative stress and arterial stiffness. However, these effects do not occur in isolation but rather within a set of physiological and pathological variables that influence vascular homeostasis.

Thus, the evaluation of methylarginines as biomarkers of endothelial function must consider the interaction between different regulatory factors. The isolated interpretation of these compounds can lead to limited conclusions, and an approach that includes the analysis of metabolic profiles and the consideration of hormonal and inflammatory factors is essential.

Finally, there is a need for future studies to deepen the understanding of the mechanisms by which methylarginines affect vascular health, exploring their relationship with different populations and physiological conditions. This research may contribute to the development of more effective strategies for the prevention and management of vascular dysfunctions associated with aging.

## Declaration of interest

The authors declare that there is no conflict of interest that could be perceived as prejudicing the impartiality of the work reported.

## Funding

This work did not receive any specific grant from any funding agency in the public, commercial, or not-for-profit sector.

## Author contribution statement

EEAC and RMSA independently performed all stages of study identification, screening, and eligibility, including reading titles, abstracts, and full texts, as well as data extraction using a standardized form. Disagreements were resolved by consensus, consulting CCDF whenever necessary. EEAC was responsible for developing the search strategy and initial draft of the manuscript. RMSA contributed to the organization of data, critical review of content, and adherence to methodological guidelines. CCDF supervised the process, contributed to the methodological design, ensured compliance with PRISMA standards, and performed the technical and scientific review of the final version. All authors approved the final version for publication.

## References

[1] Lesthaeghe R. The second demographic transition, 1986–2020: sub-replacement fertility and rising cohabitation – a global update. Genus 2020 76 10. (10.1186/s41118-020-00077-4)

[2] Oliveira AS. Demographic transition, epidemiological transition and population aging in Brazil. [Article in Portuguese] Hygeia Rev Bras Geogr Médica E Saúde 2019 15 69–79 . (10.36660/abc.20210708)

[3] Oliveira AC, Cunha PMGM, Vitorino PVDO, et al. Vascular aging and arterial stiffness. Arq Bras Cardiol 2022 119 604–615. (10.36660/abc.20210708) 36287415 PMC9563886

[4] Ungvari Z, Tarantini S, Donato AJ, et al. Mechanisms of vascular aging. Circ Res 2018 123 849–867. (10.1161/circresaha.118.311378) 30355080 PMC6248882

[5] Polanczyk CA. Epidemiology of cardiovascular diseases in Brazil: the hidden truth in numbers. Arq Bras Cardiol 2020 115 161–162. (10.36660/abc.20200793) 32876178 PMC8384285

[6] Lunkes LC, Murgas LDS, Dorneles EMS, et al. Socioeconomic factors related to cardiovascular diseases: a review. Hygeia Rev Bras Geogr Médica E Saúde 2018 14 50–61. (10.14393/hygeia142804)

[7] Melo LAD, Braga LDC, Leite FPP, et al. Factors associated with multimorbidity in the elderly: an integrative literature review. Rev Bras Geriatr E Gerontol 2019 22 e180154. (10.1590/1981-22562019022.180154)

[8] Barroso WKS, Rodrigues CIS, Bortolotto LA, et al. Brazilian guidelines for arterial hypertension – 2020. [Article in Portuguese] Arq Bras Cardiol 2021 116 516–658. (10.36660/abc.20201238) 33909761 PMC9949730

[9] Camici GG, Savarese G, Akhmedov A, et al. Molecular mechanism of endothelial and vascular aging: implications for cardiovascular disease. Eur Heart J 2015 36 3392–3403. (10.1093/eurheartj/ehv587) 26543043

[10] Gallo G & Savoia C. New insights into endothelial dysfunction in cardiometabolic diseases: potential mechanisms and clinical implications. Int J Mol Sci 2024 25 2973. (10.3390/ijms25052973) 38474219 PMC10932073

[11] Dusse LMS, Vieira LM & Carvalho MDG. Review on nitric oxide. J Bras Patol Med Lab 2003 39 [cited August 14, 2025]. (https://www.scielo.br/scielo.php?script=sci_arttext&pid=S1676-24442003000400012&lng=pt&nrm=iso&tlng=pt)

[12] Wang M, Monticone RE & McGraw KR. Proinflammatory arterial stiffness syndrome: a signature of large arterial aging. J Vasc Res 2018 55 210–223. (10.1159/000490244) 30071538 PMC6174095

[13] Vatner SF, Zhang J, Vyzas C, et al. Vascular stiffness in aging and disease. Front Physiol 2021 12 762437. (10.3389/fphys.2021.762437) 34950048 PMC8688960

[14] Ya J & Bayraktutan U. Vascular ageing: mechanisms, risk factors, and treatment strategies. Int J Mol Sci 2023 24 11538. (10.3390/ijms241411538) 37511296 PMC10380571

[15] Ma S, Xie X, Yuan R, et al. Vascular aging and atherosclerosis: a perspective on aging. Aging Dis 2025 16 33. (10.14336/ad.2024.0201-1) PMC11745439 38502584

[16] Hooijmans CR, Rovers MM, De Vries RB, et al. SYRCLE’s risk of bias tool for animal studies. BMC Med Res Methodol 2014 14 43. (10.1186/1471-2288-14-43) 24667063 PMC4230647

[17] Klawitter J, Hildreth KL, Christians U, et al. A relative L-arginine deficiency contributes to endothelial dysfunction across the stages of the menopausal transition. Physiol Rep 2017 5 e13409. (10.14814/phy2.13409) 28904082 PMC5599867

[18] McEvoy M, Schofield P, Smith W, et al. Memory impairment is associated with serum methylarginines in older adults. Curr Alzheimer Res 2014 11 97–106. (10.2174/15672050113106660178) 24156258

[19] Staniszewska A, Rajagopalan S, Al-Shaheen A, et al. Increased levels of symmetric dimethyl-arginine are associated with all-cause mortality in patients with symptomatic peripheral arterial disease. J Vasc Surg 2015 61 1292–1298. (10.1016/j.jvs.2015.01.002) 25776186

[20] Schulze F, Carter AM, Schwedhelm E, et al. Symmetric dimethylarginine predicts all-cause mortality following ischemic stroke. Atherosclerosis 2010 208 518–523. (10.1016/j.atherosclerosis.2009.06.039) 19700158

[21] Meinitzer A, Kielstein JT, Pilz S, et al. Symmetrical and asymmetrical dimethylarginine as predictors for mortality in patients referred for coronary angiography: the ludwigshafen risk and cardiovascular health study. Clin Chem 2011 57 112–121. (10.1373/clinchem.2010.150854) 21036946

[22] Schlesinger S, Sonntag SR, Lieb W, et al. Asymmetric and symmetric dimethylarginine as risk markers for total mortality and cardiovascular outcomes: a systematic review and meta-analysis of prospective studies. PLoS One 2016 11 e0165811. (10.1371/journal.pone.0165811) 27812151 PMC5094762

[23] Forstermann U & Sessa WC. Nitric oxide synthases: regulation and function. Eur Heart J 2012 33 829–837. (10.1093/eurheartj/ehr304) 21890489 PMC3345541

[24] Neves JA, Neves JA & Oliveira RDCM. Biomarkers of endothelial function in cardiovascular diseases: hypertension. [Article in Portuguese] *J Vasc Bras* 2016 15 224–233. (10.1590/1677-5449.000316)

[25] Scalera F & Bode-Böger SM. Asymmetric dimethylarginine accelerates cellular senescence. In Organizador. Tumor Dormancy, Quiescence, and Senescence, vol 2, pp 3–16. Ed MA Hayat. Dordrecht: Springer Netherlands, 2014. [cited August 14, 2025]. (https://link.springer.com/10.1007/978-94-007-7726-2_1)

[26] Ferrari-Souza JP, Brum WS, Hauschild LA, et al. Vascular risk burden is a key player in the early progression of Alzheimer’s disease. Neurobiol Aging 2024 136 88–98. (10.1016/j.neurobiolaging.2023.12.008) 38335912 PMC11416150

[27] Virarkar M, Alappat L, Bradford PG, et al. L-arginine and nitric oxide in CNS function and neurodegenerative diseases. Crit Rev Food Sci Nutr 2013 53 1157–1167. (10.1080/10408398.2011.573885) 24007420

[28] National Heart, Lung, and Blood Institute. Quality Assessment Tool for Observational Cohort and Cross-Sectional Studies. Bethesda (MD): NHLBI, 2017. (https://www.nhlbi.nih.gov/health-topics/study-quality-assessment-tools)

